# Congenital mastocytoma: clinical and histopathological aspects of a case with unusual presentation^☆^

**DOI:** 10.1016/j.abd.2026.501371

**Published:** 2026-05-13

**Authors:** Daniela da Guarda Ribeiro, Larissa Toledo de Lima Duarte Souza, Ângela Cristina Leitão de Souza, Juliany Lima Estefan

**Affiliations:** Service of Dermatology, Pediatric Dermatology Outpatient Clinic, Hospital Federal de Bonsucesso, Rio de Janeiro, RJ, Brazil

Dear Editor,

Mastocytosis constitutes a heterogeneous group of hematopoietic disorders characterized by the abnormal proliferation of mast cells in different tissues. The clinical presentation may be restricted to the skin (cutaneous mastocytosis), generally with a benign course, or involve other organs, configuring systemic mastocytosis, which may present with severe clinical manifestations.^1^ Solitary cutaneous mastocytoma represents about 10% to 15% of all pediatric cases of cutaneous mastocytosis, and among these, 60% are congenital. Typically, the age of onset occurs in childhood, especially in the first three months of life.^2^, ^3^

The present report describes a newborn female, 39 weeks, with no known family or gestational history. On initial physical examination, an erythematous plaque with brownish borders, infiltrated and hardened, with an "orange peel" appearance, well-defined borders, approximately 6 cm in its largest diameter, was observed on the posterior-lateral aspect of the left thigh (Fig. 1). At 9 days of age, there was a progressive increase in the lesion associated with infiltration, accompanied by the appearance of blisters on the plaque and the presence of Darier’s sign (Fig. 1).

**Fig. 1:**
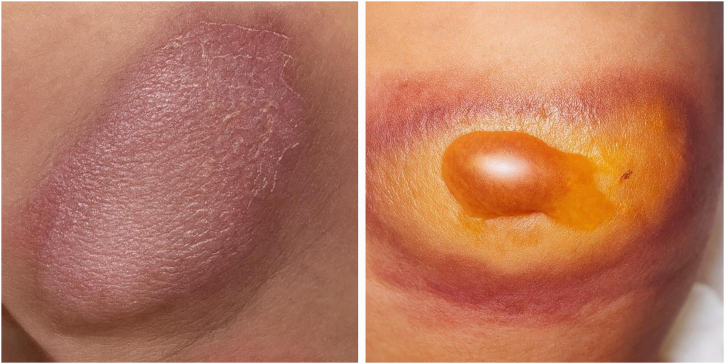
The image on the left shows the mastocytoma at birth. The image on the right shows the mastocytoma on the 9th day of life, with the appearance of blisters.

Given this clinical picture, the diagnostic hypothesis of congenital cutaneous mastocytoma was raised, with subcutaneous adiponecrosis of the newborn and juvenile xanthogranuloma being considered in the differential diagnosis, which can present with similar symptoms in infants.

Complementary tests were requested for diagnostic evaluation and exclusion of systemic mastocytosis, which, although rare, can lead to gastrointestinal and hematological disorders, with a potentially significant impact on the child’s quality of life. Skin biopsy revealed dermal proliferation of monomorphic and fusiform round cells, diffusely arranged in the papillary and upper reticular dermis (Fig. 2), with granular cytoplasm evidenced by Giemsa staining (Fig. 3). Immunohistochemistry demonstrated diffuse positivity for CD117 (c-Kit), a sensitive marker for mast cells, corroborating the diagnosis (Fig. 3).

**Fig. 2:**
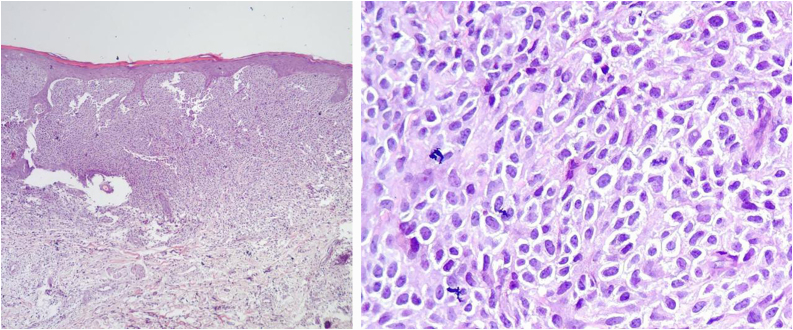
The image on the left shows monomorphic and fusiform round cells, diffusely arranged in the papillary and upper reticular dermis. On the right, at higher magnification, numerous mast cells stained with Hematoxylin & eosin are observed.

**Fig. 3:**
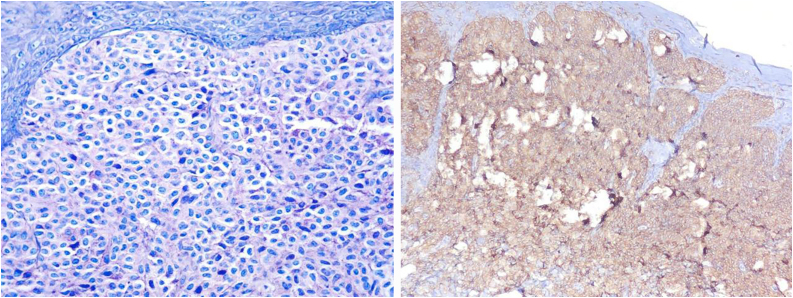
The image on the left shows mast cell proliferation using Giemsa staining. On the right, the CD117 (c-KIT) immunohistochemical reaction is diffusely positive in mast cells.

Serum tryptase levels were normal (5.31 μg/L), and abdominal ultrasound and laboratory tests (complete blood count, liver function, and renal function) showed normal results.

Due to the lesion size, excision was not performed, prioritizing clinical management. A medium-potency topical corticosteroid was prescribed as needed for symptomatic control.

Currently, the patient is two years old, with adequate neuropsychomotor development, without recurrence of blisters or systemic symptoms, in addition to gradual involution of the skin lesion (Fig. 4). She remains under regular clinical follow-up.

**Fig. 4:**
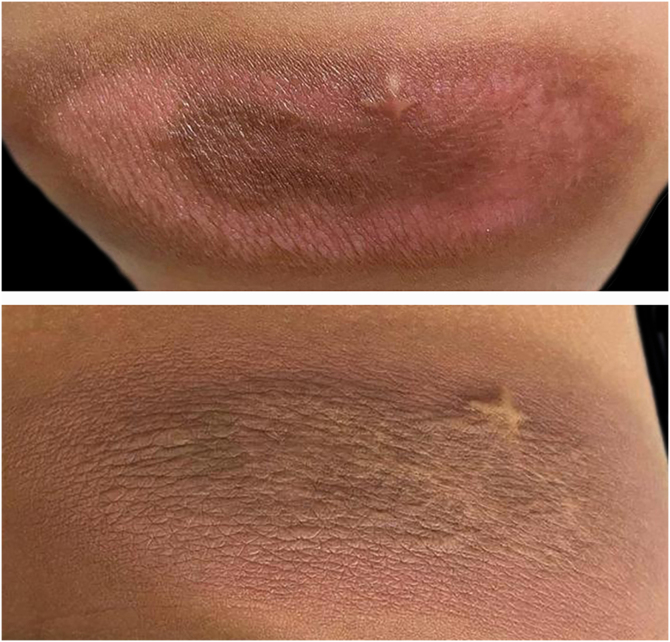
The lesion progression can be observed by comparing it at the age at nine months (left) and two years (right).

The correlation between clinical, histopathological, and immunohistochemical findings, associated with the exclusion of systemic forms and relevant differential diagnoses, was fundamental for confirming the diagnosis. The favorable evolution during the first two years of life, with gradual lesion involution and absence of systemic manifestations, reinforces the benign and self-limiting nature of the condition.

In the reported case, it is possible that the patient manifested the blister at the site of the lesion due to more intense manipulation of the region in the first days of hospital admission. This greater friction, added to the anatomical location close to the diaper area, a region subject to friction, moisture, and constant contact, may have contributed to the local triggering of the phenomenon. It is known that, in patients with cutaneous mastocytosis, several factors can act as triggers for mast cell degranulation and lesion exacerbation, including mechanical irritation (friction or massage), surgical trauma, physical exertion, stress, and extreme temperatures. In addition, external stimuli such as alcohol consumption, spicy foods, hot drinks, certain medications (aspirin, nonsteroidal anti-inflammatory drugs, antibiotics, and opioids), vaccines, and even iodinated radiocontrast agents are also described as potentially triggering.^3^, ^4^ Thus, the combination of local friction and repeated manipulation may have acted as a precipitating factor in this case.

Solitary mastocytoma usually has an excellent prognosis, even with a positive Darier sign for years and the presence of blisters in the initial phase. In most cases, remission is achieved by adulthood, without transition to systemic mastocytosis.^5^

Early recognition of congenital mastocytoma is extremely important, not only to establish the correct diagnosis and avoid unnecessary procedures, but also to guide families regarding the natural progression of the disease, possible complications, and preventive measures related to mast cell degranulation. Furthermore, ruling out malignant conditions or systemic mastocytosis directly contributes to the safe management of the condition.^3^

## Authors’ contributions

Daniela da Guarda Ribeiro: Design and planning of the study; data collection; drafting and editing of the manuscript; effective participation in research orientation; intellectual participation in the propaedeutic and/or therapeutic conduct of the studied cases; critical review of the literature; approval of the final version of the manuscript.

Larissa Toledo de Lima Duarte Souza: Effective participation in research orientation; intellectual participation in the propaedeutic and/or therapeutic conduct of the studied cases; critical review of the literature; approval of the final version of the manuscript.

Ângela Cristina Leitão Souza: Effective participation in research orientation; intellectual participation in the propaedeutic and/or therapeutic conduct of the studied cases; critical review of the literature; approval of the final version of the manuscript.

Juliany Lima Estefan: Effective participation in research orientation; intellectual participation in the propaedeutic and/or therapeutic conduct of the studied cases; critical review of the literature; approval of the final version of the manuscript.

All authors approved the final version of the manuscript and are fully responsible for the published content.

## Financial support

None declared.

## Research data availability

Does not apply.

## Conflicts of interest

None declared.
